# Differences in non-replacement dairy calf health and sale price between calves sold at livestock auctions and directly to calf raisers

**DOI:** 10.3168/jdsc.2026-1080

**Published:** 2026-07-27

**Authors:** K.C. Creutzinger, S. Albers, R. Steiner

**Affiliations:** 1 Department of Animal and Veterinary Sciences, College of Agriculture and Life Sciences, University of Vermont, Burlington, VT 05405; 2 School of Veterinary Medicine, University of Wisconsin–Madison, Madison, WI 53706; 3 Lodi Veterinary Clinic, Lodi, WI 53555

## Abstract

Nonreplacement dairy calves are commonly sold via auction or directly to calf purchasers during early life and may be managed differently on the dairy depending on their route of sale. Thus, the objectives of this study were to investigate whether calf health and sale price differed between routes of sale and whether calf health status influenced sale price. A total of 212 nonreplacement dairy calves were enrolled in this cross-sectional study at a single livestock auction (n = 106) and 6 dairy farms (n = 106) in central Wisconsin. Calves sold through an auction were assessed at the auction (n = 63) and on their farm of birth before sale (n = 43) and all calves sold directly were assessed at their farm of birth before sale (n = 106). Calves were evaluated for signs of dehydration, diarrhea, and respiratory disease, and a blood sample was collected to assess failed transfer of passive immunity (FTPI). Sale price was recorded for all calves, and calves sold at auction were weighed. Calves sold at auction were more likely to have FTPI and diarrhea than those sold directly. The average sale price was not different between calves sold via auction or directly. However, sale price at auction was associated with calf weight and calves sold directly had set prices. At auction, breed but not sex or health status were associated with sale price. Although this study had a limited sample size, it identified that calf health was different at the time of sale between auction and direct sale.

## Graphical Abstract

**Figure:**
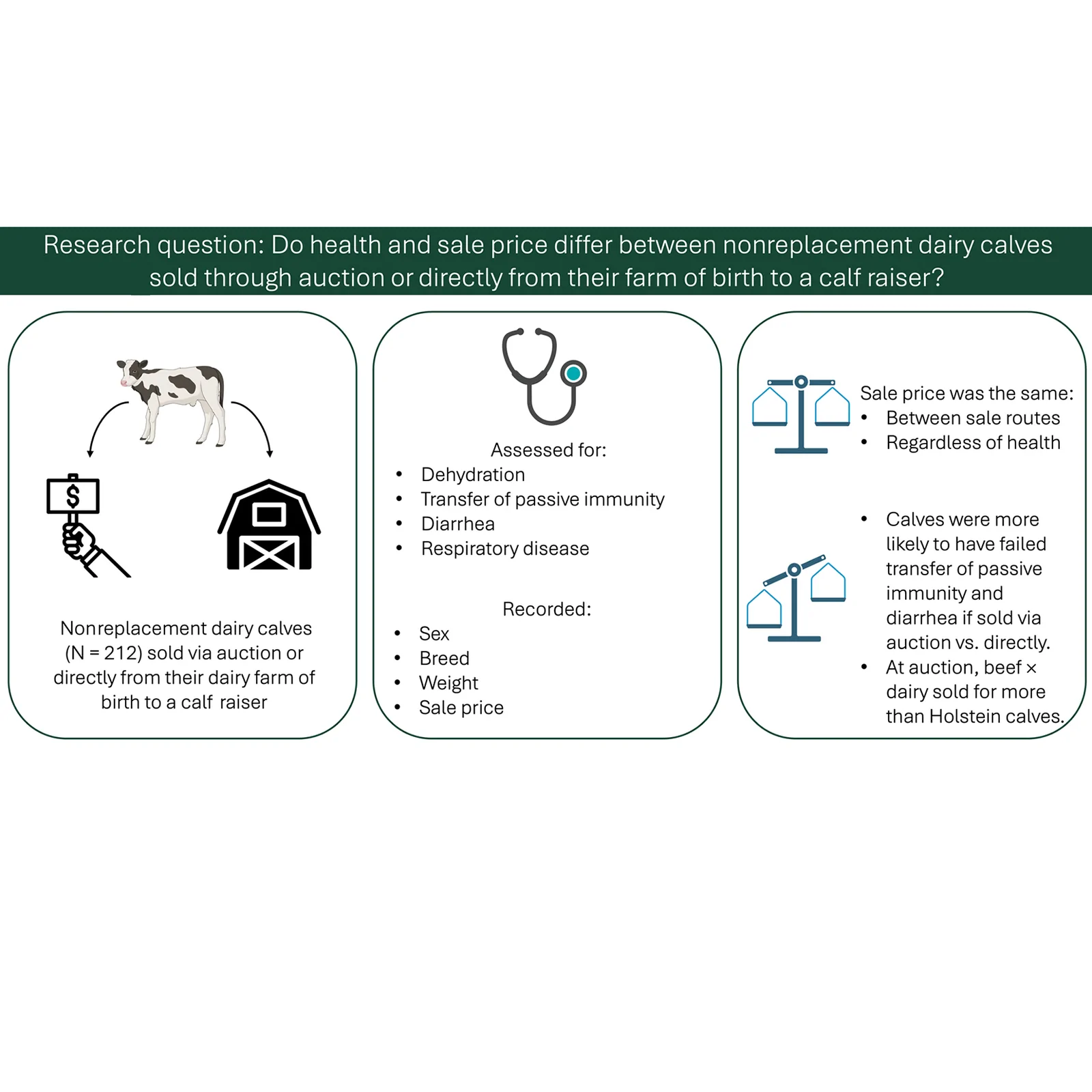

A portion of calves born on dairy farms are not retained to maintain the lactating herd and are referred to as nonreplacement dairy calves. In the United States, nonreplacement dairy calves have unique welfare challenges compared with those in other global markets because they are typically sold from their farm of birth within a week of age (Machuca et al., 2026). In other countries, calf sales during early life are prohibited; for example, Canada requires that calves are at least 9 d of age to be sold via auction (Government of Canada, 2019) and sale from farms of birth in other countries is limited by transportation laws; in the European Union and Germany, for example, transportation of calves less than 14 and 28 d of age, respectively, is prohibited (European Council, 2005; EFSA AHAW Panel et al., 2022). Calf sales during early life require that we understand the relationship between calf sales and their welfare.

Nonreplacement dairy calves face several welfare challenges from birth to arrival at a calf raiser, such as suboptimal care on the farm of birth, long periods of time without feed and water, and comingling with calves from multiple sources (Creutzinger et al., 2021). Some challenges for calves, including hunger and dehydration, can be easily treated. However, challenges such as suboptimal colostrum may affect calves throughout their lives by increasing their likelihood of morbidity and mortality (Renaud et al., 2020b; Schinwald et al., 2022).

Nonreplacement dairy calf health is important to production (Schinwald et al., 2022) and calf welfare (reviewed by EFSA AHAW Panel et al., 2023) and may differ at the point of sale depending on the sale route. Renaud et al. (2018) and Maggard et al. (2024) found that nonreplacement dairy calves had greater mortality and diarrhea, respectively, when sold via a livestock auction or calf dealer compared with calves sold directly from the farm. Consistent with these results, a focus group of dairy producers in the Midwestern United States reportedly provided poorer care to nonreplacement than replacement calves, including suboptimal quality colostrum and housing (Creutzinger et al., 2022). Furthermore, the participants felt a stronger obligation to provide optimal care and to keep nonreplacement calves healthy before sale if the calves were sold directly to a calf raiser than through an auction due to the direct connection with the calf buyer. Because of the complex nature of the calf supply chain in the United States, the relationship between sale routes and health requires further investigation.

The sale of nonreplacement calves has historically generated a small amount of revenue for dairy producers, but has increased in recent years (USDA, 2026). As the sale prices of nonreplacement calves increase, calf raisers have a greater economic risk if their return on investment is lost or diminished from calf morbidity or mortality. However, it is unclear if the risk of poor health and weight gain during the rearing period is reflected in the calf sale price.

The objective of this study was to investigate whether calf health and sale price differ between livestock auctions and direct farm to calf raiser sales, and to determine if calf sale price is affected by calf health. We hypothesized that calves would be in better health and sell for a greater amount if they were sold directly from a dairy producer to a calf raiser than if they were sold through a livestock auction. Additionally, we hypothesized that calves would sell for a lower amount at auction if they had diarrhea or respiratory disease.

A cross-sectional observational study was performed at one livestock auction (central, WI) and 6 dairy farms (south-central, WI) from June to August 2023. The livestock auction was selected based on its proximity to the researchers and willingness to participate. Dairy farms were recruited based on their relationship with the herd veterinarian, if they sold some or all nonreplacement dairy calves directly to a calf raiser, and their willingness to participate. Participants were not remunerated for their participation in the study.

Newborn nonreplacement calves (n = 212) were enrolled in the trial during a single visit to a livestock auction (n = 63) and during weekly visits to dairy farms (n = 149). Calves enrolled during dairy farm visits were either sold directly to calf raisers (n = 106) or through livestock auctions (n = 43; Figure 1). Sample size considerations were based on detecting differences in the prevalence of failed transfer of passive immunity (**FTPI**) between calves sold via auction and directly at α = 0.05 and β = 0.80, with final enrollment determined by calf availability.

**Figure 1:**
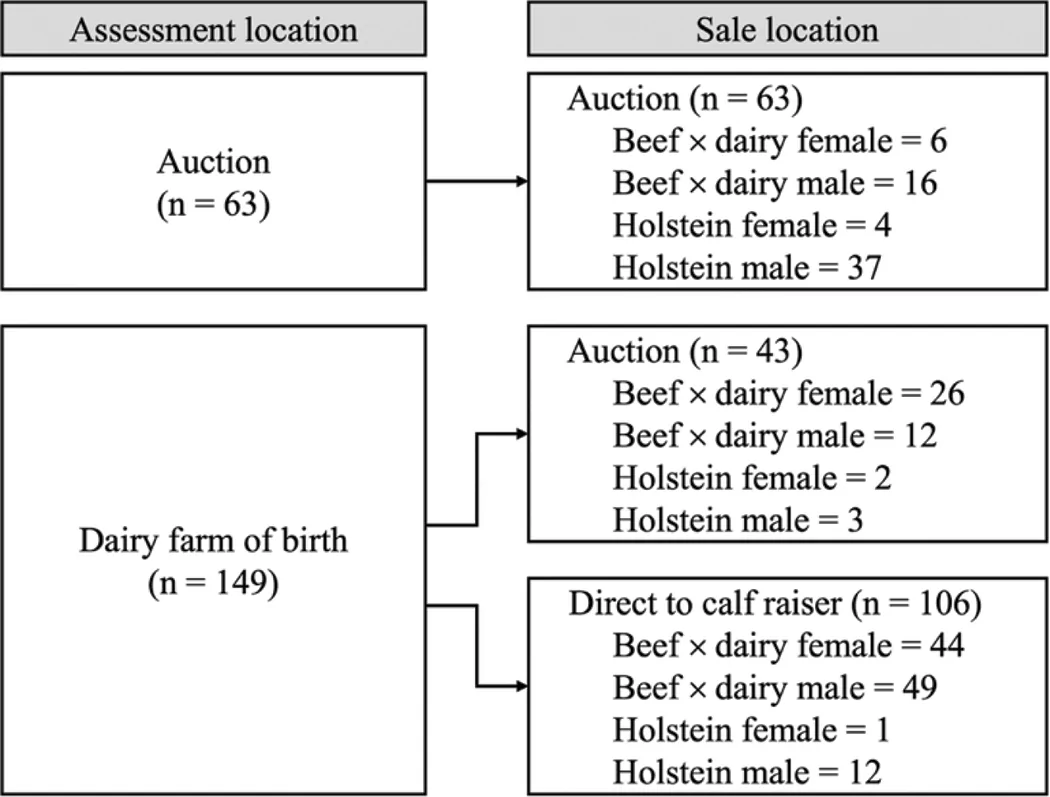
Descriptive statistics of nonreplacement dairy calves (n = 212) sold directly from dairy farms (n = 106) or through a livestock auction (n = 106) in central Wisconsin, by sex and breed.

Health assessments and blood collections were performed on the day of sale for all calves. Calves enrolled at livestock auctions were assessed at the auction, and calves enrolled at their farm of birth were assessed before leaving the farm. Calf sex (male or female) and breed (Holstein or beef × dairy) were recorded for all calves. Age (d) was recorded for calves assessed at dairy farms. Calf age, farm care information, and duration of transportation to the sale barn for calves enrolled at the auction were not available. On sale days, calves were dropped off at the auction in the morning (between 0600 and 1000 h) and housed in group holding pens with calves from several farms; feed and water were not available. At the time of sale, individual calves were walked through an arena and sold to the highest bidder, then sorted into group pens by purchaser.

Health assessments were performed before calf sales. Fecal consistency was visually observed from spontaneous defecation or gently stimulating the rectum with a thermometer (0 = normal, firm but not hard; 1 = soft, does not hold form, piles but spreads slightly; 2 = runny, spreads readily; 3 = watery, liquid consistency, spatters; Renaud et al., 2020a). Diarrhea was recognized as a fecal score ≥2. Respiratory score was assessed (nasal discharge, eye discharge, presence of an ear droop or head tilt, presence of cough, elevated rectal temperature ≥39.5°C, and rapid or labored breathing; Love et al., 2014) and considered abnormal if the total score was ≥5. Dehydration was assessed via a skin tent test (0 = returns to normal in <2 s, 1 = returns to normal in 2 s, 2 = returns to normal in 2–4 s, 3 = returns to normal in 5–8 s, 4 = returns to normal in >8 s; Smith, 2009). Calves were considered dehydrated if the skin tent score was ≥2. Assessments were performed by authors KC and SA with high inter-rater reliability for all scored outcomes (>90% agreement).

Blood samples were taken within 1 h after sale for calves assessed at the auction (n = 50) and at the time of health assessment for calves enrolled on the dairy of birth (n = 149) to assess serum total protein (**STP**) concentration. Some calves (n = 13) were unable to be blood sampled because of severe dehydration. Samples were not excluded based on calf age (4 d of age: n = 24, 5 d of age: n = 1), despite being outside of the optimal age range for evaluation of STP (24 to 48 h after birth; Goetz et al., 2025). Calf age was not available for calves sold at auction, but calves were expected to range from 1 to 7 d of age (Machuca et al., 2026). Thus, removing samples based on age only from calves assessed at the farm may have biased the STP results for this population only. Blood was collected from each calf via jugular venipuncture using a 20-gauge, 2.54-cm needle into a 10-mL sterile blood collection tube without anticoagulant (BD Vacutainer; Becton, Dickson and Co., Franklin Lakes, NJ). Blood was allowed to clot at room temperature for 60 to 120 min, then centrifuged at 1,500 × *g* for 15 min at approximately 20°C. Serum was collected, and STP concentration was determined using a clinical refractometer (National Industry Supply, Temecula, CA). A cut-point of <5.1 g/dL was used to define FTPI (Lombard et al., 2020).

Calf BW (kg) was recorded for all calves sold through a livestock auction at the time of sale using the scales available at the auction. Weight was not recorded for directly sold calves. The sale price of each calf ($US) was recorded.

Data were entered into Microsoft Excel (Microsoft Corp., Redmond, WA) and manually checked for errors and completeness. All statistical analyses were performed using SAS (version 9.4, SAS Institute Inc., Cary, NC).

Prevalence estimates for poor health outcomes, including dehydration, FTPI, diarrhea, and respiratory disease, were calculated as the total number of calves with the poor health outcome divided by the number of calves sampled within the specified category. The SURVEYFREQ procedure (SAS) was used to produce prevalence estimates.

To evaluate associations between dichotomized dependent health outcomes and independent variables related to calf characteristics (e.g., sex, breed, dehydration, diarrhea) and routes of sale, univariable analysis for each dependent variable was performed. Independent variables with moderate statistical association (*P* < 0.2) were included in multivariable analysis. Backward stepwise manual elimination was performed for multivariable models by removing variables with the highest *P*-value until all *P*-values in the model were *P* < 0.20. Calf assessment and sale location were retained in all models regardless of significance. Potential confounders were tested to determine if removal changed the model by at least 20% and were retained if so (Dohoo et al., 2009). No confounders were identified. Model fit was assessed by visual assessment of residual versus fitted and quantile-quantile residual plots for continuous outcomes and Pearson chi^2^/df for binary outcomes. Significance was declared at *P* ≤ 0.05 and tendencies at *P* ≤ 0.10.

The odds of occurrence of poor health outcomes, including dehydration, FTPI, diarrhea, and respiratory disease were assessed with a generalized linear model (PROC GLIMMIX) with a binary distribution and logit link. Calf assessment location (auction vs. dairy farm of birth) and sale route (auction vs. direct) were combined into a single variable to assess the effect of assessment location and sale route: auction assessment-sale at auction, dairy farm assessment-sale at auction, and dairy farm assessment-direct to purchaser sale. The dehydration model included diarrhea, sex, breed, and assessment location-sale route as independent variables; assessment location-sale route was included in the final model. The FTPI model included sex, breed, dehydration status, and assessment location-sale route as independent variables; assessment location-sale route was included in the final model. The diarrhea model included sex, breed, FTPI, and assessment location-sale route as independent variables; assessment location-sale route was included in the final model. The respiratory disease model included sex, breed, FTPI, and assessment location-sale route as independent variables; assessment location-sale route was included in the final model.

The difference in calf sale price between direct sale and auction was assessed using a *t*-test (PROC TTEST). Effects of predictor variables on calf sale price were only analyzed for calves sold at auction due to the flat rate pricing of calves purchased directly. A linear model (PROC MIXED) was used to assess factors associated with sale price. Univariable analysis tested calf sex, breed, diarrhea, cough, nasal discharge, eye discharge, ear droop, dehydration, FTPI, respiratory disease, and weight as predictor variables. The final multivariable model included calf breed and weight as independent variables.

A total of 212 calves were enrolled in this study: 63 were assessed at the auction at the time of sale, 43 were assessed at their dairy farm of birth before being sold through an auction, and 106 were assessed at their farm of birth before they were sold directly to a calf raiser. Of the 6 enrolled farms, 2 sold calves exclusively to direct purchasers, 3 sold calves either directly to a calf raiser or through an auction, and one sold calves exclusively through an auction. Four of the farms sold to a single calf raiser, and one sold calves to 3 different calf raisers; one calf raiser purchased calves from 2 of the farms (n = 15 calves).

Calves sold via auction weighed 41.5 ± 5.9 kg (mean ± SD; range: 27.2–59.0 kg; beef × dairy female = 39.9 ± 6.3 kg, beef × dairy male = 44.0 ± 5.6 kg, Holstein female = 35.9 ± 2.7 kg, Holstein male = 41.9 ± 5.2 kg). Of the calves assessed at the farm of birth, age at the time of sale was 2.8 ± 0.9 d (range: 1–4 d) and 2.2 ± 1.4 d (range: 1–5 d) for calves sold via auction and directly, respectively.

Dehydration prevalence was 32.1% (34/106) and 19.8% (21/106) for calves sold via auction and directly, respectively. Of the calves sold at auction, 33.3% of calves (21/63) assessed at the auction were dehydrated compared with 30.2% of calves (13/43) assessed at the farm of birth. There was a tendency (*P* = 0.053) for dehydration to be affected by assessment and sale location (auction assessment and sale vs. dairy assessment and direct sale, odds ratio [**OR**]: 2.5, 95% CI: 1.2–5.2; auction assessment and sale vs. dairy assessment and auction sale, OR: 1.4, 95% CI: 0.6–3.4).

The average STP for calves sold via auction was 5.81 ± 0.93 g/dL and directly to calf raisers was 5.86 ± 0.67 g/dL (Figure 2). Failed transfer of passive immunity prevalence was 25.8% (24/93) and 9.4% (10/106) in calves sold via auction and directly to calf raisers, respectively. Of the calves sold via auction, 22.0% (11/50) of calves assessed at the auction and 30.2% (13/43) of calves assessed at their farm of birth had FTPI. The odds of FTPI were influenced by assessment and sale location (*P* < 0.01). Calves were more likely to have FTPI if sold via auction and assessed at an auction (OR: 2.7, 95% CI: 1.1–6.9, *P* = 0.04) or dairy (OR: 4.2, 95% CI: 1.6–10.5, *P* = 0.003) than if sold directly. There was no difference in the likelihood of FTPI between calves sold through an auction and assessed at an auction or dairy (OR: 1.5, 95% CI: 0.6–3.9, *P* = 0.37).

**Figure 2:**
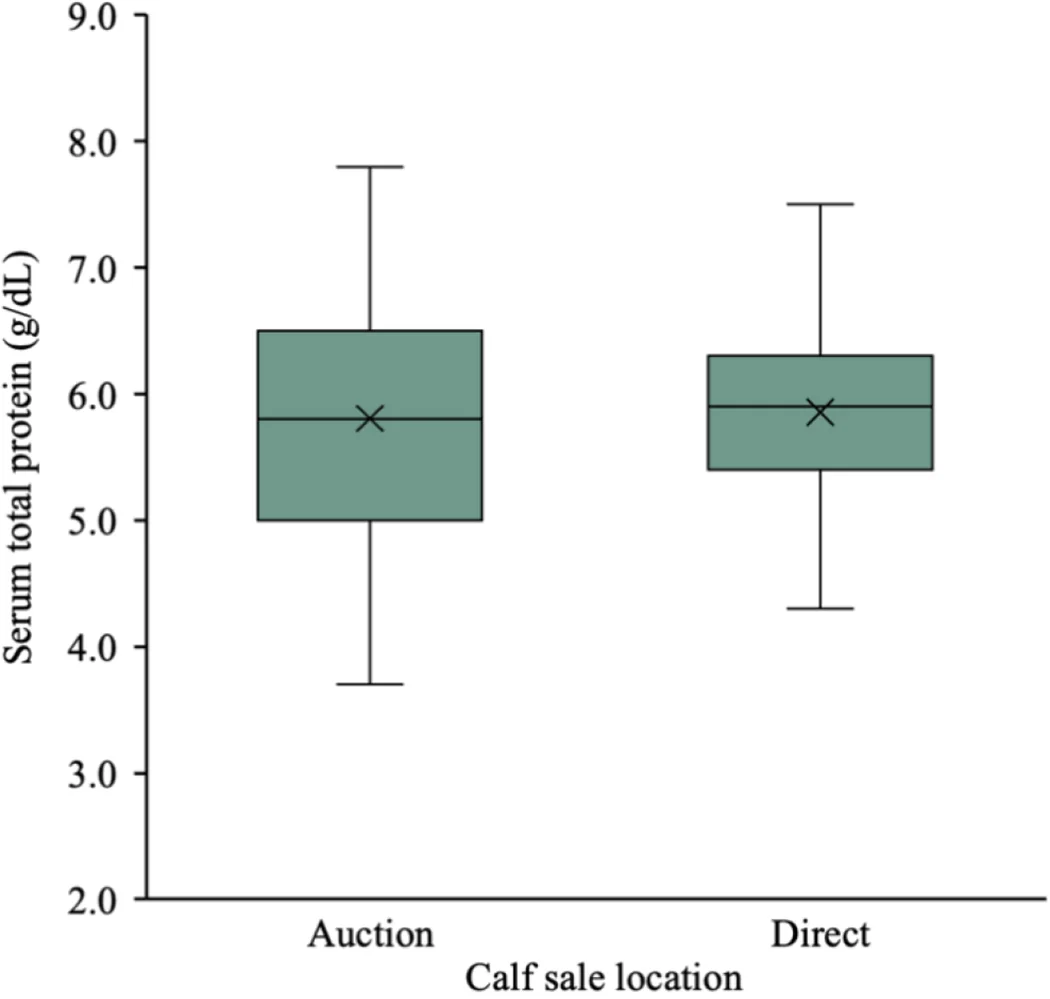
Box and whisker plot of STP (g/dL) of nonreplacement dairy calves sold through auction (n = 93) or directly from their farm of birth (n = 106). Calves were assessed at a single auction and on 6 dairy farms in central Wisconsin. For calves assessed at auction, blood samples were taken after sales were conducted (n = 50), and calves assessed on their farm of birth were blood sampled on the day of sale before leaving the farm (n = 149). The line in the middle of each box represents the median value, the “×” shows the mean value, the edges of the boxes show the 25th (bottom) and 75th (top) percentiles, and the thin lines (whiskers) represent the price range.

Diarrhea prevalence was 23.6% (25/106) and 8.5% (9/106) for calves sold via auction and directly, respectively. Of the calves sold at auction, the diarrhea prevalence was 33.3% (21/63) and 9.3% (4/43) for calves assessed at auction and at the farm of birth, respectively. The likelihood of diarrhea was affected by the combination of assessment and sale location (*P* < 0.001). Calves were more likely to have diarrhea if they were assessed and sold at the auction than if they were assessed at the dairy and sold at auction (OR: 4.9, 95% CI: 1.5–15.6, *P* = 0.007) or if they were assessed at the dairy and sold directly (OR: 5.4, 95% CI: 2.3–12.8, *P* < 0.001). There was no difference in the likelihood of diarrhea between calves assessed at the dairy of birth and sold directly or through an auction (OR: 1.1, 95% CI: 0.3–3.8, *P* = 0.87).

Respiratory disease prevalence was 13.2% (14/106) and 9.4% (10/106) for calves sold via auction and directly, respectively. Of the calves sold at auction, 7.9% (5/63) assessed at the auction had respiratory disease compared with 20.9% (9/43) assessed at the farm of birth. The likelihood of respiratory disease was not different between the combination of sale and assessment location (*P* = 0.11).

Dairy farms sold calves directly to 6 different calf purchasers for prices that varied by purchaser, including $200 regardless of calf sex or breed (11/106), $500 for beef × dairy calves only (3/106), $320 regardless of sex or breed (71/106), $400 for only female beef × dairy calves (2/106), a range of $250 to $400 for beef × dairy calves regardless of sex or breed (13/106) but received 1 beef × dairy freemartin female calf for $0, and $250 for male Holstein calves (4/106). Average sale price for calves sold directly and at auction was $302.6 ± $63.3 (range: $0–$500; Figure 3) and $316.1 ± $156.8 (range: $5–$600), respectively. There was no difference in average sale price between calves sold at auction and directly to calf raisers (t-value = −0.8, *P* = 0.4).

**Figure 3:**
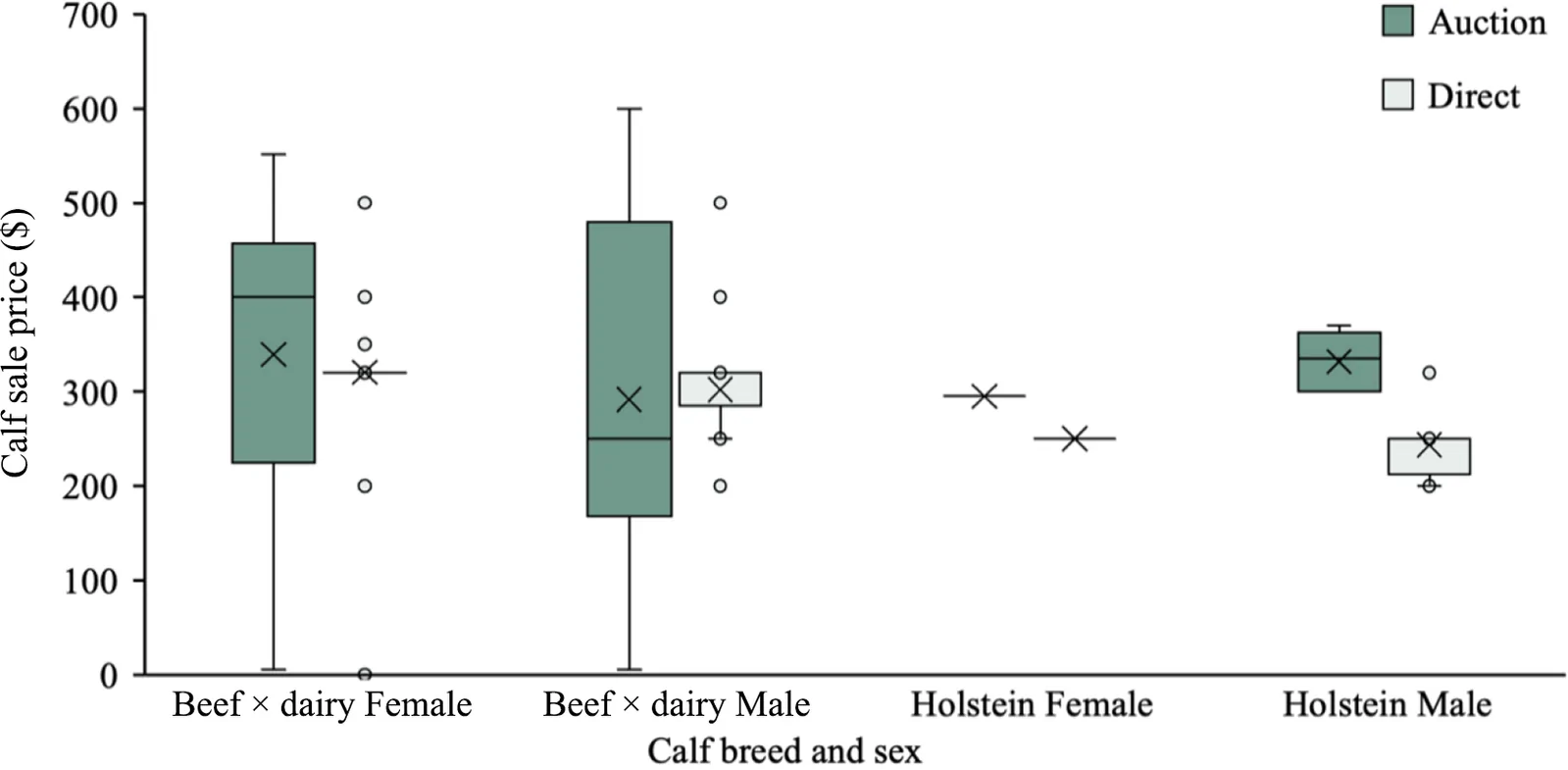
Box and whisker plots of the sale price of nonreplacement dairy calves sold during early life at a livestock auction (n = 106) or directly from their farm of birth to calf purchasers (n = 106). Calves were assessed at a single auction and on 6 dairy farms in central Wisconsin. Calf sale prices are distributed by breed and sex. The line in the middle of each box represents the median value, the “×” shows the mean value, the edges of the boxes show the 25th (bottom) and 75th (top) percentiles, the thin lines (whiskers) represent the price range, and the dots are outliers.

Sale price for calves sold at auction was affected by calf weight (*F*_1,101_ = 59.4, *P* < 0.0001) and breed (*F*_1,101_ = 47.3, *P* < 0.0001). The amount calves sold for increased as calf weight increased (intercept = −297.2, slope ± SE = 14.0 ± 1.8). Beef × dairy crossbred calves sold for more than Holstein calves (LSM ± SE = $377.8 ± $14.2 vs. $230.9 ± $15.9, respectively; *P* < 0.001). Calf sex was not associated with sale price (*P* = 0.19).

Calves were more likely to have FTPI if they were sold through an auction than directly from the dairy farm to calf raisers (25.8% vs. 9.4% of calves, respectively). Current recommendations suggest that less than 10% of dairy calves should have FTPI (Lombard et al., 2020). The prevalence of calves with FTPI that were sold directly to calf raisers met the suggested cut-point. However, calves sold via auction had FTPI 2.5 times higher than the recommended benchmark. Differences in FTPI between calves sold directly and through auctions could be attributed to differences in colostrum management. Previous studies in the United States and Canada have found that nonreplacement dairy calves are sometimes given lower volumes of colostrum, poorer quality, and delayed feedings compared with replacement heifer calves (Shivley et al., 2019; Creutzinger et al., 2022). Renaud et al. (2020b) also found that male calves had lower STP concentration than heifer calves when both were assessed on their farm of birth. However, Kern et al. (2025) found that the majority dairy farms in their study in Indiana and Michigan had the same colostrum management practices for all calves regardless of breed or sex. Greater FTPI in auction compared with direct-sale calves could also be related to a difference in calf age. Ages were not available for calves assessed at auction, but previous research has found that most calves are sold through auction between 1 to 7 d of age (Kern et al., 2025) and calves sold directly were 1 to 5 d old. The optimal window to assess STP is 24 to 48 h after birth as IgG concentration declines from 3 to 7 d of life (Goetz et al., 2025). However, this relationship is unclear as calves sold at auction and assessed at the farm of birth were sold between 1 to 4 d of age and had a higher prevalence of FTPI than calves assessed and sold at auction. Additional research to investigate differences in colostrum management of nonreplacement dairy calves prior to sale by auction or direct to calf raisers is warranted.

Calves assessed at auction were more likely to have diarrhea than those assessed at their dairy of birth (23.6% vs. 8.5% of calves, respectively). The prevalence of diarrhea in this study falls within the range of previously reported prevalences for diarrhea of nonreplacement dairy calves assessed at a livestock dealer in Ohio (13.1%; Maggard et al., 2024) and upon arrival to a calf raiser in Canada (48.3%; Schafer et al., 2026). However, the relationship between calf source and diarrhea is inconsistent. One study found that calves were less likely to have diarrhea if they were sourced from a third party than directly from a farm (Maggard et al., 2024). Comparatively, Schinwald et al. (2022) found that male nonreplacement dairy calves at a calf raiser had more days with diarrhea if they were sourced from a drover (i.e., an individual that collects calves from multiple farms and transports them to veal facilities) compared with calves purchased directly from farms, but there was no difference for calves sourced from auctions. Calf source may not be as influential on calf health as FTPI. Schafer et al. (2026) found that calves were less likely to have diarrhea if they had successful transfer of passive immunity (**TPI**) and were more likely to have successful TPI if they were sourced from farms compared with from a calf dealer. Diarrhea is an important welfare concern because calves with diarrhea are more likely to have depression (Schafer et al., 2026), indicative of a negative welfare state, and it is a leading cause of death during the milk feeding period at calf raisers (Schinwald et al., 2022). Differences in the prevalence of calf diarrhea between sale route should be further explored in other regions, such as the European Union and Germany, which require that calves are at least 14 and 28 d of age, respectively, before transportation from the farm of birth (European Council, 2005; EFSA AHAW Panel et al., 2022). Diarrhea prevalence in nonreplacement dairy calves increases from 0 to 1 wk and decreases from 1 to 3 wk postarrival to a calf raiser (Schinwald et al., 2022), which could influence calf attitude and the presence of diarrhea at the point of sale depending on calf age.

A total of 11.6% of calves in this study had respiratory disease. In a similar study, Maggard et al. (2024) found that very few calves had signs of respiratory disease at a livestock dealer (6.7% ocular discharge, 1.2% ear droop, 0.6% nasal discharge). However, another study in the Midwestern United States found that upon arrival to a nonreplacement calf raiser, 11.8% of calves had respiratory disease and beef × dairy crosses were more likely to have respiratory disease than Holstein calves (Schafer et al., 2026). Similar to diarrhea, more research is needed to understand the causes of respiratory disease in young nonreplacement dairy calves due to its widespread occurrence and impact. In a prospective cohort study at a calf raiser, 71% of nonreplacement dairy calves were treated for respiratory disease with antibiotics, and it accounted for 59.2% of deaths during the milk feeding period (Schinwald et al., 2022). The aforementioned study highlights the negative effect of respiratory disease on calf mortality, making it an important area of future research in nonreplacement calves.

Approximately one-third of calves were dehydrated before sale, which is similar to the prevalence of dehydrated calves upon arrival to a calf raiser reported in previous studies in the United States and Canada (32%, Scott et al., 2020; 35.8%, Schafer et al., 2026, respectively). Calves tended to have greater odds of dehydration if they were assessed at the auction compared with calves assessed at their farm of birth. A study of nonreplacement dairy calves found that calves were more likely to be dehydrated if they were transported for 12 or 16 h compared with 6 h (6 h: 13%, 12 h: 47%, 16 h: 54%; Goetz et al., 2023). Based on the results from Goetz et al. (2023), it appears as though calves in this study were without water for at least 6 h. This is likely true for the calves sold at auction because water was not provided in the holding pens. Water availability on dairies was not recorded, but it is possible that the calves did not have continuous access to water; approximately only half of farms in the United States provide water to calves immediately before transportation (Machuca et al., 2026). Calf dehydration at the point of sale should be considered globally because transportation regulations vary. In the United States, calves can be transported for up to 28 h without stopping (National Archives and Records Administration, 1974); by comparison, Canada limits transportation to 12 h for calves less than 8 d of age (Government of Canada, 2019), the European Union requires rest and water for calves after 9 h of travel (European Council, 2005), and Australia limits transportation to 6 h for calves less than 5 d of age and requires water after 18 h for 5- to 30-d-old calves (Animal Health Australia, 2009). Thus, the prevalence and severity of dehydration of calves at the point of sale is likely to vary globally and should be explored regionally.

By the time calves are clinically dehydrated, they have already likely been experiencing prolonged thirst, which is detrimental to welfare. The high rates of dehydration observed in this study at auction and at the dairy farm of birth highlights that efforts should be made to provide calves with sufficient drinking water to avoid dehydration throughout calf rearing and along the supply chain.

Data in this study were collected in 2023. At the time of data collection, calves, on average, were sold for slightly over $300 through auctions and directly from dairy farms. Since then, the nonreplacement dairy calf market has changed substantially, with calves between 0 to 14 d of age currently selling for more than $1,000 for males and $400 to $800 for females (USDA, 2026). Contrary to our hypothesis, health was not associated with sale price at auctions. At auctions, calves that weighed more and had beef × dairy genetics sold for more than lighter calves and straightbred dairy breeds. The difference in sale price between calf breeds is consistent with previously published literature (Wilson et al., 2020; Buczinski et al., 2021). Interestingly, calves sold through auctions and those marketed directly from dairy farms did not differ in average sale price. However, purchasers who bought calves directly from farms had set prices per calf dependent on sex and breed, while auction prices at auction had greater individual variability. For some producers and calf buyers, stable pricing from direct calf sales may offer greater predictability compared with variable auction markets (Creutzinger et al., 2022; Cheng et al., 2024). We recommend that future research explores calf purchaser attitudes toward dairy calves and what influences their purchasing decisions. These data may provide insight to support better calf outcomes before sale.

This study has several limitations. Data were collected at 1 livestock auction on 1 sale day, limiting the sample size, and dairy farm enrollment relied on convenience sampling. Although this study provides novel insight into nonreplacement dairy calf sales in Wisconsin, it is not representative of the entire industry or changes over time. Another limitation is that BCS was not recorded. Anecdotally, the authors noted that many calves were underconditioned at the livestock auction, but it was not part of the formal assessment.
